# Cell and tissue polarity in the intestinal tract during tumourigenesis: cells still know the right way up, but tissue organization is lost

**DOI:** 10.1098/rstb.2013.0014

**Published:** 2013-11-05

**Authors:** Aliya Fatehullah, Paul L. Appleton, Inke S. Näthke

**Affiliations:** Cell and Developmental Biology, University of Dundee, Dundee DD1 5EH, UK

**Keywords:** adenomatous polyposis coli, polarity, adhesion, migration, tissue organization, organoid culture

## Abstract

Cell and tissue polarity are tightly coupled and are vital for normal tissue homeostasis. Changes in cellular and tissue organization are common to even early stages of disease, particularly cancer. The digestive tract is the site of the second most common cause of cancer deaths in the developed world. Tumours in this tissue arise in an epithelium that has a number of axes of cell and tissue polarity. Changes in cell and tissue polarity in response to genetic changes that are known to underpin disease progression provide clues about the link between molecular-, cellular- and tissue-based mechanisms that accompany cancer. Mutations in adenomatous polyposis coli (APC) are common to most colorectal cancers in humans and are sufficient to cause tumours in mouse intestine. Tissue organoids mimic many features of whole tissue and permit identifying changes at different times after inactivation of APC. Using gut organoids, we show that tissue polarity is lost very early during cancer progression, whereas cell polarity, at least apical–basal polarity, is maintained and changes only at later stages. These observations reflect the situation in tumours and validate tissue organoids as a useful system to investigate the relationship between cell polarity and tissue organization.

## 1. Introduction

Tissue polarity is a key feature of epithelia, which line all cavities in the body [1]. Epithelia are the site for most human cancers, and the loss of tissue organization found in malignancies is usually accompanied by loss of polarity, although the degree of change and the stage at which this loss occurs varies with tumour and tissue type.

**Figure 1.**
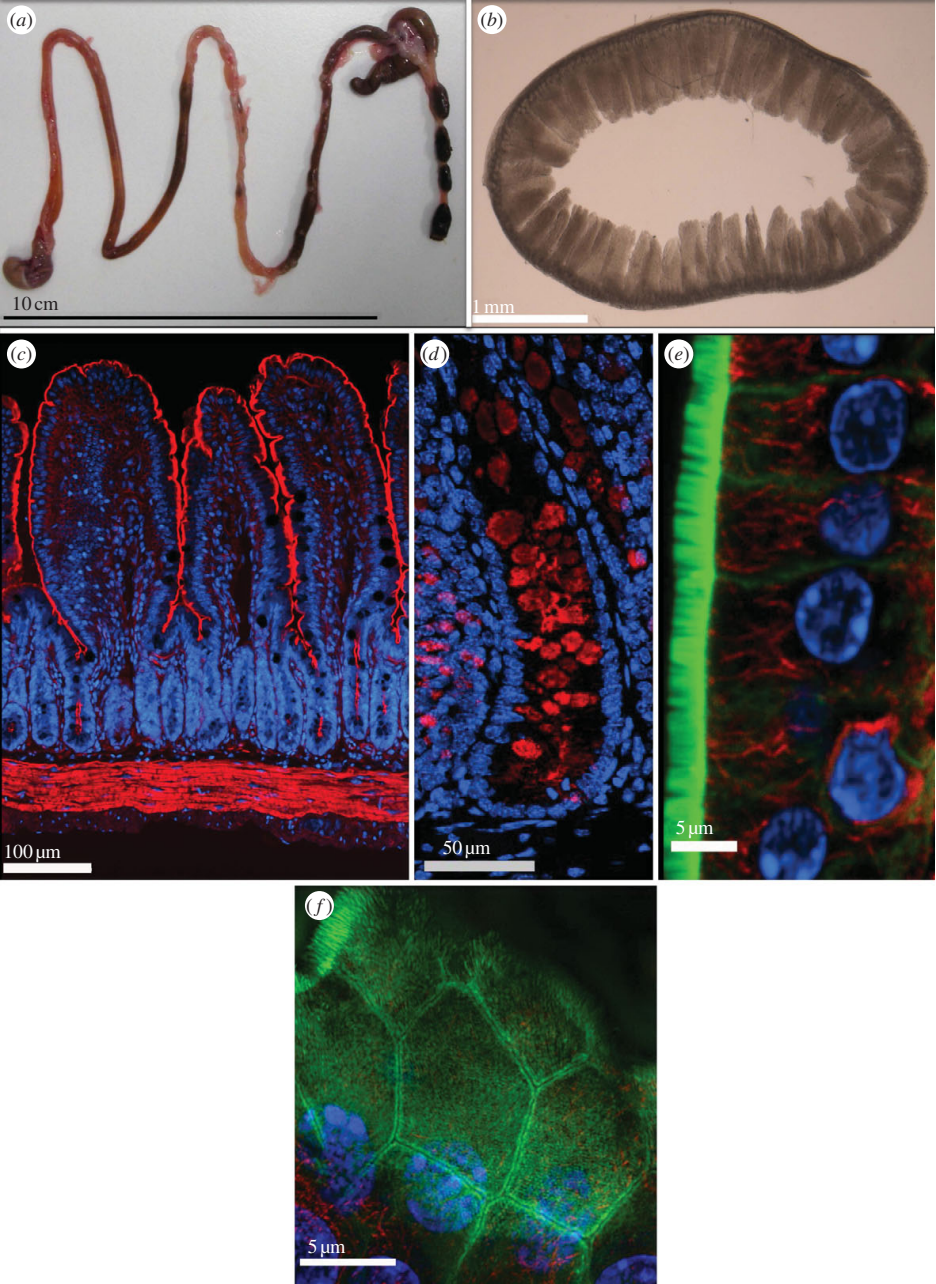
Gut tissue organization at different levels of resolution. (*a*) The entire mouse gut from the stomach (bottom left) to the rectum (bottom right) is shown. The caecum (top right corner) marks the transition from small to large intestine. (*b*) A 200 µm thick transverse section through the mouse duodenum with villi protruding into the lumen. (*c*) Mouse small intestine stained with phalloidin to show F-actin (red) and Hoechst 33342 to show nuclei (blue) reveals crypts at the base of finger-like villi and above a layer of muscle cells rich in F-actin. (*d*) Mouse colonic crypt stained with Hoechst 33342 to show nuclei and anti-mucin antibodies to show goblet cells (red). (*e*) A confocal optical section through mouse small intestinal epithelium stained with phalloidin to show F-actin (green) and anti-tubulin antibody to reveal the microtubule (red) arrangement in the cells of the crypt and Hoechst 33342 to show nuclei (blue). (*f*) Maximum intensity projection of an image stack acquired with an OMX super resolution microscope, showing F-actin (green), microtubules (orange) and nuclei (blue) in small intestinal epithelial cells. Using super resolution allows separation of the plasma membranes of neighbouring cells.

We use the intestinal tract, specifically the small and large intestine, to understand how changes in the polarity contribute to disease. The intestinal tract epithelium is characterized by a number of axes of polarity that reflect different levels of resolution (figure 1). Like most simple epithelia, gut epithelium displays strong apical–basal polarity with a highly developed, F-actin-rich brush border in the apical plasma membrane of absorptive epithelial cells and a similarly highly polarized organization of the other cell types that populate this tissue (figure 1*c–e*). The other main lineages in the gut epithelium are secretory cells, specifically Paneth, goblet and enteroendocrine cells (figure 1*d*) [2–6]. They secrete antimicrobial peptides, mucus and hormones, respectively, from their apical surface. Paneth cells reside at the bottom of intestinal crypts where they help to create the niche for stem cells, whereas goblet and enteroendocrine cells are distributed throughout the epithelium at different densities in different parts of the intestinal tract [2,3].

The epithelium itself is further organized into specific tissue structures that display polarity. Tissue invaginations called crypts of Lieberkühn are directly adjacent to villi in the small intestine and in the colon open into the lumen [4]. These crypts house stem cells at their U-shaped base that provide the progenitors for cells that are continuously discarded from the villi or the crypt collar [4,7]. The axis from crypt to villus (small intestine) or crypt to lumen (colon) distinguishes cells closer to the gut wall from those closer to the lumen. Whether this axis along which cells differentiate and migrate has molecular features of planar cell polarity (PCP) such as the planar-polarized distribution of specific proteins is not known, but the directional migration and differentiation along this axis suggests that elements of PCP may operate. PCP factors such as Frizzled are expressed and are present in the gut epithelium and may contribute to the organization of niches created by Wnt signalling [8,9].

Additional axes of tissue organization exist in this tissue. A longitudinal tissue axis (proximal–distal) runs from the stomach to the rectum and the organization of the tissue changes along with the different physiological functions of each segment. In the small intestine, villi increase the surface area of the intestinal wall to ensure highly efficient nutrient absorption. Along the small intestine, the length and density of villi vary [10]. The colon, where packing of waste material and water resorption is achieved, lacks villi. The relative proximity of the colonic wall to the inside or outside of the peritoneal cavity creates another axis that may also correlate with proximity to the mesenteric circulation and enervation.

A characteristic and important feature of gut epithelium is its constant renewal. Absorptive cells have an average lifespan of 3–5 days in the intestine and are constantly replenished from stem cell pools. This translates into 20–50 million cells being lost per minute in humans creating the most highly turning over tissue in the body [10]. This incredibly high turnover relies on continued and rapid cell proliferation throughout life and makes the occurrence of tumourigenic mutations extremely likely. So it is not surprising that mutations that lead to colorectal cancer (CRC) are particularly common in the elderly and that CRC is the second most common cause of cancer deaths in the UK and the third most common cancer in the Western World (http://www.cancerresearchuk.org/cancer-info/cancerstats/types/bowel/mortality/ and http://www.cancerresearchuk.org/cancer-info/cancerstats/types/bowel/incidence/uk-bowel-cancer-incidence-statistics#geog).

The molecular changes that underpin CRC are well characterized. Importantly, mutations in a single gene, the *adenomatous polyposis coli* (*Apc*) gene, are common to almost all CRCs [11]. These mutations can be detected in the earliest stages of CRC that can be diagnosed. The high penetrance of APC mutations in gut epithelium, in particular, make understanding the function of the APC protein in the processes that govern normal gut tissue homeostasis vitally important. Understanding how loss of normal APC contributes to tissue organizational and particularly polarity changes can inform about the contribution of different cellular processes to cell and tissue polarity and the relationship between them.

## 2. Studying polarity and adhesion in the gut epithelium

High-resolution imaging and detailed biochemical- and cell-based approaches have revealed much about the state of adhesion and polarity in epithelia. However, most of the studies involving tissue rely on snapshots available in fixed samples of sectioned tissue or averages measured in populations of cells. To understand the relationship between different cellular processes and their impact on individual components of the cellular machinery that governs polarity and adhesion requires the ability to monitor the dynamics of cells in the relevant tissue. The advent of gut organoid cultures that mirror many features of tissue *in situ* have improved our ability to understand how different cellular processes effect tissue dynamics [12]. They can be biochemically and genetically manipulated and are more readily observed as a whole unit than tissue *in situ* and allow observation of single cells.

**Figure 2.**
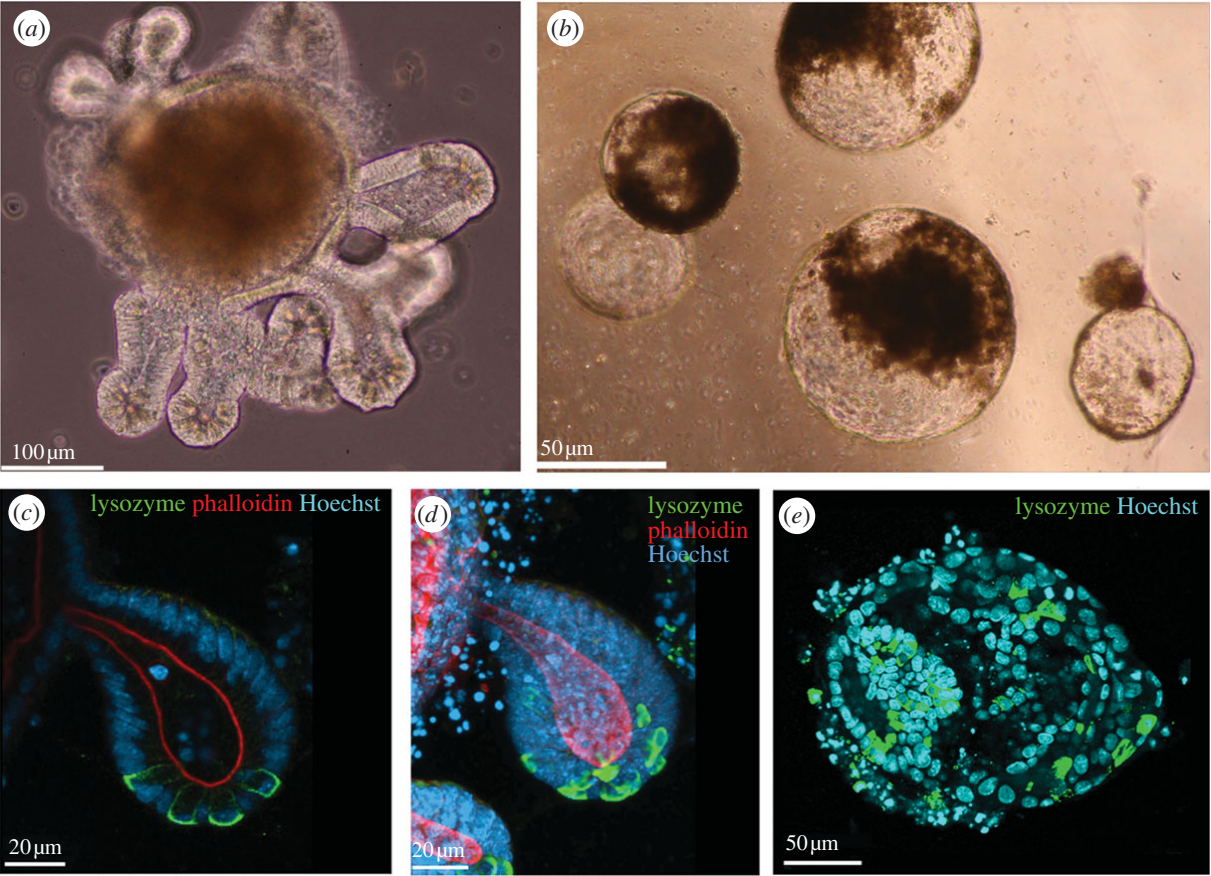
Loss of APC results in altered tissue organization in organoids. Phase contrast images of intestinal organoids in Matrigel show (*a*) the highly branched morphology of a mature, wild-type organoid that mimics tissue organization *in situ* and (*b*) the cyst-like structures formed by tissue isolated from intestinal tissue lacking APC. The dark patches visible on the cysts are created by dying cells that were shed into the lumen of the cyst as part of the normal cellular turnover. Paneth cells are arranged in an alternating pattern at the base of the branched crypt-like structures in wild-type organoids shown in a confocal optical section (*c*) and maximum intensity projection (*d*) in a wild-type organoid stained with anti-lysozyme antibodies (green), phalloidin to show F-actin (red) and Hoechst 33342 to mark the nuclei (blue). (*e*) In APC^*fl/fl*^ organoids, Paneth cells are no longer clustered to a specific region and are not arranged in an alternating pattern but are distributed randomly throughout the organoid stained with anti-lysozyme antibody to mark Paneth cells (green) and Hoechst 33342 to mark the nuclei (blue).

Gut organoids can be grown in Matrigel using isolated stem cells or whole intestinal crypts as starting material [12]. They develop crypt-like structures that contain stem and Paneth cells, and are connected to regions of non-dividing cells that appear to form a regular, differentiated epithelium reminiscent of that in villi (figure 2*a*). They contain the full complement of cell types normally found in the epithelium although it is not clear yet whether their relative abundance is similar to that in tissue [13].

Here, we show that changes in polarity that accompany cancer in this tissue, at least at the early stages, affect tissue polarity rather than cell polarity. Early adenoma maintain apical–basal polarity judged by the normal appearing brush border and cell junctions. However, the axis of differentiation along the crypt–villus axis is altered. This not only raises important questions about the relationship between cell and tissue polarity, but also illustrates that intestinal and colonic epithelium are an excellent model system to study this relationship and its molecular basis.

## 3. Method and materials

### (a) Tissue preparation

All experiments involving animals were performed under the UK Home Office guidelines. Wild-type and *APC^fl^*^/*fl*^ *Cre* CL57BL/6 mice were killed by cervical dislocation, and small intestine was removed immediately. The tissue was washed and immersed into cold fixative containing 4% paraformaldehyde at pH 7.4 overnight at 4°C before processing for staining [14].

### (b) Crypt isolation protocol

Organoids were generated from isolated intact crypts, from mouse small intestine as described previously [12]. Briefly, small intestine was removed immediately after cervical dislocation and washed with cold PBS. The tissue was incubated with 3 mM EDTA in PBS followed by mechanical shaking to remove crypts. The crypt suspension was then centrifuged at 4°C for 3 min at 600 rpm and washed with PBS to remove villi and single cells. The suspension was then passed through a 70 µm cell strainer (BD Biosciences). The resulting pure crypt suspension was then placed in growth factor reduced phenol-free Matrigel (BD Biosciences). Crypt medium (advanced DMEM/F12 supplemented with HEPES, glutamax, *N*-acetylcycysteine, N2 and B27) along with R-Spondin, Noggin and EGF was added to the culture. For passaging, organoid cultures were washed with cold advanced DMEM/F12 medium, and Matrigel and organoids were broken up mechanically by pipetting. After further washes to remove dead cells, organoid suspensions were mixed with 100 µl of Matrigel and placed into 24 well plates. The cultures were then overlaid with growth factor supplemented medium.

### (c) Preparing organoid cultures for immunofluorescence

For immunofluorescent labelling and imaging, organoids were grown in Matrigel on eight-chamber μslides (Ibidi, Martinsried, Germany). Organoids were fixed in warm fixative containing 4% paraformaldehyde (pH 7.4) for 1 h, permeabilized using permeabilization buffer (PBS, 1% triton X-100) and blocked with blocking buffer (PBS, 1% BSA, 3% normal goat serum, 0.2% Triton X-100). They were then incubated with the primary antibodies against the following antigens diluted in a working buffer (PBS, 0.1% BSA, 0.3% normal goat serum, 0.2% Triton X-100): β-catenin (1 : 300, mouse monoclonal, BD Biosciences), ZO1 (1 : 200, rabbit polyclonal, AbCam), lysozyme (to stain Paneth cells) (1 : 100, rabbit polyclonal, Zymed, Invitrogen and 1 : 2000, rabbit polyclonal, Dako), phospho-histone 3 (1 : 250, rabbit polyclonal, AbCam), Na/K ATPase (1 : 100, mouse monoclonal, AbCam), β4 integrin (1 : 100, rat monoclonal, Abcam), Ki67 (1 : 200, rabbit monoclonal, AbCam), ezrin (1 : 200, rabbit polyclonal), E-cadherin (1 : 100, rabbit polyclonal, Cell Signaling Technology) overnight at room temperature. Organoids were washed and incubated overnight in working buffer at room temperature with the appropriate Alexa Fluor secondary antibody (1 : 500) from Molecular Probes, Invitrogen; along with Hoechst 33342 (1 : 250) to counterstain nuclei and/or rhodamine phalloidin (1 : 250, Invitrogen) to stain F-actin. Organoids were washed with working buffer and mounted using ProLong gold antifade (Molecular Probes, Invitrogen). Organoids were imaged with a Zeiss 710 confocal microscope, using the 40× LD plan-Neofluar objective lens, Z stacks were taken at 1 µm steps and imported to Volocity (Perkin Elmer) for analysis.

For imaging using the OMX-structured illumination microscope (Applied Precision, Issaquah, WA; figure 1*e*), images were acquired using a UPlanSApochromat 100× 1.4 NA, oil immersion objective lens (Olympus, Center Valley, PA) and back-illuminated Cascade II 512 × 512 electron-multiplying charge-coupled device camera (Photometrics, Tucson, AZ) on the OMX version 3 system equipped with 405-, 488- and 593-nm solid-state lasers. Samples were illuminated by a coherent scrambled laser light source that had passed through a diffraction grating to generate the structured illumination by interference of light orders in the image plane to create a three-dimensional sinusoidal pattern, with lateral stripes approximately 0.2 µm apart. The pattern was shifted laterally through five phases and through three angular rotations of 60° for each Z-section, separated by 0.125 µm. Exposure times were typically between 100 and 200 ms, and the power of each laser was adjusted to achieve optimal intensities of between 2000 and 4000 counts in a raw image of 16-bit dynamic range, at the lowest possible laser power to minimize photobleaching. Raw images were processed and reconstructed to reveal structures with greater resolution [15] implemented on Softworx, v. 6.0 (Applied Precision). The channels were then aligned in *x*, *y* and rotationally using predetermined shifts as measured using 100 nm TetraSpeck (Invitrogen) beads with the Softworx alignment tool (Applied Precision, Inc.). In this case, images were imported into Imaris (Bitplane, Switzerland).

## 4. Results

### (a) Using organoids as a model to study tissue polarity changes

The advent of gut organoid cultures, which exhibit many features of tissue *in situ*, has improved our ability to understand tissue dynamics [12]. Comparing organoids prepared from mice with floxed APC alleles before and after (APC*^fl/fl^*) inactivation of APC [16] shows distinct differences in organoids formed from epithelial crypts lacking APC (figure 2). Organoids from wild-type tissue exhibit a distinct branching morphology, while organoids generated from APC*^fl/fl^* tissue form single cyst-like spheroids without any branches (figure 2*b*).

To determine how the distribution of normally present cell types are affected by the altered tissue organization in the APC*^fl/fl^* organoids, we first used lysozyme to detect the localization of Paneth cells [12] (figure 2*c*,*d*). In wild-type organoids, Paneth cells clustered at the bottom of crypts in an alternating manner, reminiscent of the arrangement of Paneth and stem cells at the base of crypts in the intestine *in situ* [17]. Using phalloidin to visualize F-actin reveals the apical surface of cells and outlines the lumen of organoids to show their branched morphology. In APC*^fl/fl^* organoids, Paneth cells no longer clustered in the specific regions and did not form an alternating pattern. Instead, they appeared to be randomly scattered throughout the cyst wall (figure 2*e*). F-actin, on the other hand, still localized apically to mark the lumen of cysts, outlining their spherical shape.

**Figure 3.**
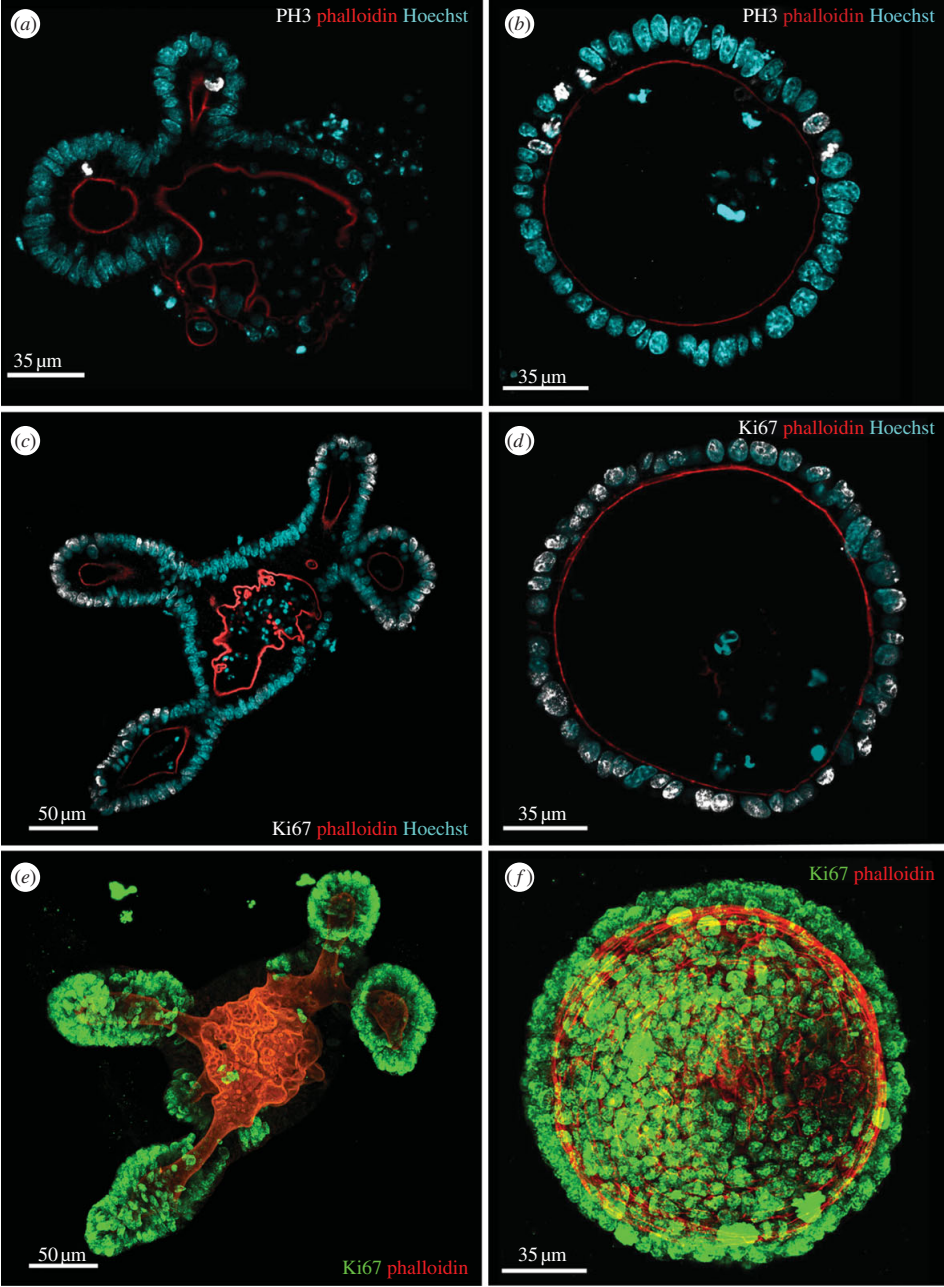
Loss of APC causes loss of distinct proliferative zones. Mitotic cells are limited to crypt-like domains of wild-type organoids usually just above the Paneth cells, but are distributed randomly in APC^*fl/fl*^ organoids. Wild-type (*a,c,e*) or APC^*fl/fl*^ (*b,d,f*) organoids were stained with antibodies against phospho-Histone 3 (*a,b*) to mark mitotic cells (white), and Ki67 (white, *c*,*d*; green, *e*,*f*) to mark proliferating cells. F-actin was visualized with rhodamine phalloidin (red) and nuclei were detected with Hoechst 33342 (blue). Optical sections (*a–d*) or maximum intensity projections (*e,f*) show a distinct loss of separate proliferative zones in the APC^*fl/fl*^ organoids.

In wild-type organoids, the branched structures are connected to regions of non-dividing cells that appear to form a regular, differentiated epithelium reminiscent of that in villi. To determine the distribution of mitotic cells in organoids, we immunostained organoids with antibodies against phospho-histone 3. Mitotic cells were limited to the crypt-like domain just above Paneth cells, similar to what is observed in whole tissue. Mitotic cells were never observed in the differentiated zone of organoids (figure 3*a*). In APC*^fl/fl^* organoids, mitotic cells were distributed randomly throughout the organoid (figure 3*b*).

To determine whether the position of dividing cells reflected the position of proliferating cells, we examined the staining pattern of Ki67. In wild-type organoids, Ki67 staining was limited to crypt domains, whereas in APC*^fl/fl^* organoids, Ki67-positive cells were distributed uniformly across the organoid and even included Paneth cells (figure 2*c–f*). Ki67-positive or mitotic Paneth cells were never observed in wild-type organoids.

Together, these results suggest that loss of APC leads to loss of the crypt–villus differentiation axis and causes a change in tissue architecture. Importantly, the APC-deficient cysts are highly reminiscent of cysts commonly present in adenoma confirming that organoids in culture recapitulate features of tissue *in situ* and provide an excellent model system to study changes in polarity during tumour progression [18].

### (b) Loss of adenomatous polyposis coli and its effects on cell polarity

**Figure 4.**
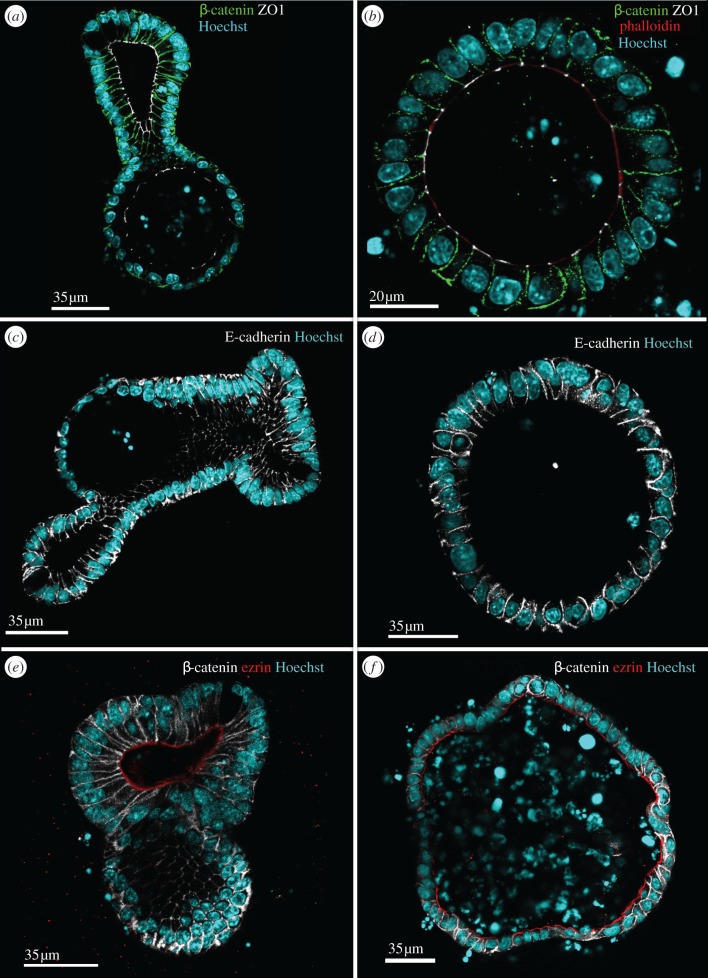
Loss of tissue polarity in APC^*fl/fl*^ organoids does not correlate with loss of apical–basal polarity. Localization of β-catenin and ZO1 to basolateral membranes and apical junctions respectively is similar in both wild-type and APC^*fl/fl*^ organoids. Wild-type (*a,c,e*) and APC^*fl/fl*^ (*b,d,f*) organoids were stained with antibodies against β-catenin (green, *a*,*b*; white, *e*,*f*) or ZO1 (white, *a,b*) or E-cadherin (white, *c,d*) and ezrin (red, *e,f*), phalloidin (red, *b*) and Hoechst 33342 to mark the nuclei (blue). Overall, the relative distribution and abundance of these polarity markers was not altered in the APC^*fl/fl*^ organoids suggesting that apical–basal polarity was not affected.

Tissue polarity is frequently altered in tumours consistent with our observation that loss of APC causes a remarkable loss of tissue organization in organoids. Loss of cell polarity also accompanies tumour development frequently. To determine whether this was the case in the distinctly different tissue morphology and organization induced by loss of APC, we used our organoid model to assess cell polarity. We first examined the staining pattern for β-catenin and ZO1 (figure 4*a,b*). APC is well known for its ability to regulate the availability of β-catenin, specifically the pool of β-catenin that regulates the activity of TCF/Lef transcription factors and not the pool of β-catenin that is bound to E-cadherin to mediate cell–cell adhesion [19]. ZO1 marks tight junctions and is involved in the maintenance of the integrity of the epithelial layer by sealing the spaces between cells, once cell–cell adhesion has been established [20]. In wild-type organoids, β-catenin localized to cell membranes where it plays a crucial role as a component of the adherens junction complex. The distribution and abundance of β-catenin was similar throughout the organoid with no obvious differences between proliferating cells in the crypt-like branches and differentiated cells in the villus-like regions. Similarly, ZO1 was evenly distributed along the differentiation axis in wild-type organoids (figure 4*a*). It concentrated at the apically localized tight junctions. In some cells, ZO1 appears along the entire apical surface (figure 4*a,b*). This impression was created by optical sectioning directly through the contact between two neighbouring cells. In APC*^fl/fl^* organoids, the distribution patterns of β-catenin and ZO1 were remarkably similar to those in wild-type organoids (figure 4*b*) with β-catenin still predominantly membrane-bound and ZO1 restricted to apical junctions.

Next, we compared the distribution of E-cadherin in wild-type and APC*^fl/fl^* organoids (figure 4*c,d*). In wild-type organoids, E-cadherin was uniformly distributed along the basal–lateral domains and this was similar along the crypt–villus axis. In APC*^fl/fl^* organoids, E-cadherin was also restricted to the basal–lateral domains of the membrane.

Although F-actin was similarly distributed and highly enriched at apical membranes in the brush border, irrespective of the presence or absence of APC (figure 3*a,b*), the normally polarized distribution of proteins could still be affected by lack of APC. To determine whether we could detect changes in brush border-associated proteins, we examined the distribution of ezrin (figure 4*e,f*). Ezrin is an apically enriched ERM (ezrin, radixin, moesin) family member protein that links plasma membrane proteins with F-actin [21,22]. N-terminal domains in ezrin directly bind to plasma membrane proteins such as the Na^+^/H^+^ exchange regulatory factor 1 (NHERF) and other PDZ-containing transmembrane proteins [23,24], whereas C-terminal regions can directly bind F-actin [25,26] or indirectly interact with actin via adaptor proteins such as PIP2 and EBP50 [27,23]. Ezrin is the only ERM family member expressed in mouse intestinal epithelium. It is required for the localization and function of the apical membrane protein NHERF, which supports normal intestinal function by maintaining the proton gradient across the membrane [28]. Additionally, ezrin depletion in mice intestine results in defective villi and mislocalization of other apical polarity markers [28]. We found ezrin localized to the apical membranes of cells in both wild-type and APC*^fl/fl^* organoids, consistent with the idea that interactions between F-actin and the plasma membrane are unaltered by lack of APC.

**Figure 5.**
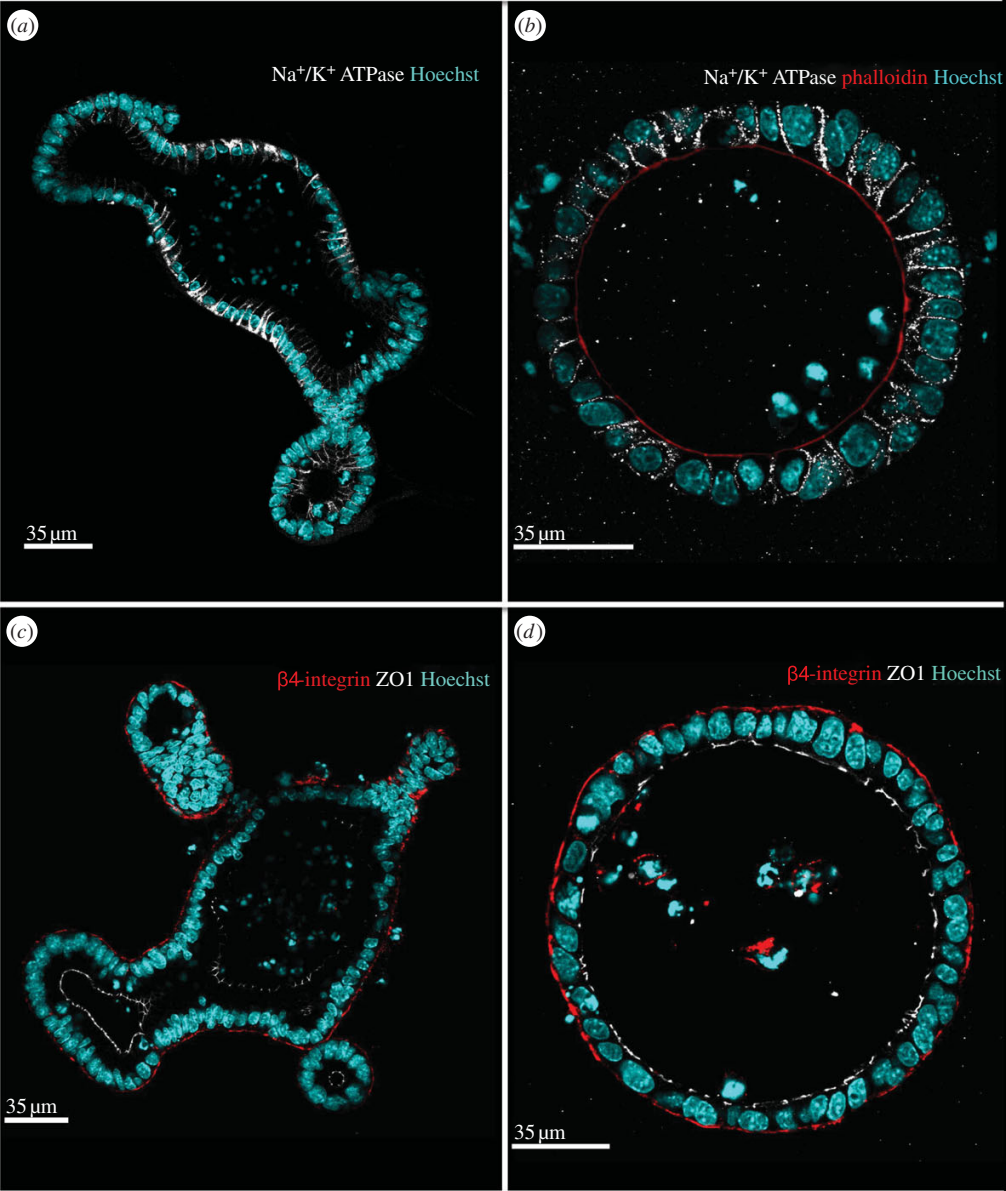
Localization of basal and lateral proteins is not altered in APC^*fl/fl*^ organoids. Wild-type (*a,c*) and APC^*fl/fl*^ (*b,d*) organoids were stained with antibodies against Na^+^/K^+^ATPase (white, *a,b*) or β4-integrin (red, *c,d*) and ZO1 (white, *c,d*), phalloidin (red, *b*) and Hoechst 33342 to mark nuclei (blue). The images showing APC^*fl/fl*^ organoids were also stained with rhodamine phalloidin. The lateral localization of the Na^+^/K^+^ATPase is maintained in both wild-type and APC^*fl/fl*^ organoids as was the basally restricted distribution of β4-integrin. In the differentiated villus-like domain the signal for the Na^+^/K^+^ATPase staining is stronger than in the crypt-like structures. The APC^*fl/fl*^ organoids exhibit a lower level of Na^+^/K^+^ATPase that is similar to the crypt-like domains in the wild-type organoids.

To examine the distribution of an additional physiologically important basal/lateral membrane protein, we determined the distribution of Na^+^/K^+^-ATPase in organoids (figure 5*a,b*). The Na^+^/K^+^-ATPase is an ion pump that belongs to the P- type ATPase family and is involved in generating the electrochemical gradient of Na^+^ and K^+^ across plasma membranes of animal cells. It consists of α- and β-subunits that bind Na^+^ and K^+^, and the β-subunit facilitates the counter-transport of K^+^ [29–32]. In wild-type organoids, Na^+^/K^+^-ATPase was distributed along lateral cell membranes throughout the organoid. Notably, it was more strongly expressed in the differentiated villus domains than in the crypt-like branches. This suggests that the differentiated epithelium of the villus requires more Na^+^/K^+^-ATPase activity to maintain the electrochemical gradient. In APC*^fl/fl^* organoids, the intensity of Na^+^/K^+^-ATPase staining was weaker than in the differentiated regions of wild-type organoids but was comparable with that in crypt-like branches of wild-type organoids. Nonetheless, it was restricted to the lateral domains, just like in wild-type cells.

The composition of the extracellular matrix (ECM) is a key contributor to the microenvironment of cells. Interactions between the ECM and cells are mediated by membrane receptors such as integrins [33–35]. In intestinal cells, α6β4 integrin functions as a specific receptor for laminins [36–38]. To elucidate how organoids interact with the ECM and whether this is affected by lack of APC, we compared the distribution of the β4 integrin in wild-type and APC*^fl/fl^* organoids (figure 5*c,d*). In wild-type organoids (figure 5*c*), β4 integrin was exclusively localized to the base of cells. Similarly, in APC*^fl/fl^* organoids (figure 5*d*), β4 integrin was limited to the basal domain of the membrane.

## 5. Discussion

The gut organoid model has gained popularity as an *in situ* model to study tissue dynamics since it was first described by Sato and co-workers in 2009. The model allows for the observation of gut epithelium in isolation, without influence from other tissues, such as muscle for instance, which surrounds crypts, and immune cells, which are abundant throughout villi as they scan the epithelium for pathogens that have breached the epithelial barrier. Here, we use this model to identify changes in tissue and cell architecture during tumourigenesis in three dimensions using the well-documented loss of APC as a tumour-initiating event.

The effects of APC depletion or loss on the intestine have been described previously [39]. That lack of APC leads to formation of round cysts with loss of distinct crypt–villus-like structures in the organoid model has also been reported [40]. However, a detailed comparison of the organization and distribution of different cell types in APC wild-type and mutant organoids is not available. We found that lack of APC results in a change from a clustered to a random distribution of Paneth and also of PH3-positive, mitotic cells. This correlated with a loss of distinct zones of proliferative and non-proliferative cells with most cells proliferating.

The relationship between lack of crypt–villus tissue polarity and apical–basal cell polarity has not been discussed at depth. The organoid model permits studying changes in cell polarity with improved imaging resolution. The localization of β-catenin, which marks the adherens junctions, was relatively unaffected by loss of APC and we did not detect relocalization of β-catenin to the nucleus in APC^*fl/fl*^ organoids, perhaps indicating that APC-independent functions of β-catenin are largely responsible for the tissue changes observed in the APC*^fl/fl^* organoids [41]. The loss of APC did not impact on tight junction assembly and formation as indicated by the remarkably similar distribution of ZO1 in wild-type and APC*^fl/fl^* organoids (figure 4*a,b*), with the protein limited to the cell–cell junctions near apical membrane. A similar result was obtained when we examined adherens junctions by analysing E-cadherin localization in organoids (figure 4*c,d*). In wild-type organoids, E-cadherin staining was evenly distributed in the lateral membrane of cells and was evenly present in all regions of the organoid. In APC*^fl/fl^* organoids, E-cadherin was also restricted to the basolateral membrane but there consistently was a higher background to signal ratio. This could indicate a reduced level of expression of E-cadherin in the APC*^fl/fl^* organoids, consistent with previous findings that APC positively affects expression of E-cadherin and contributes to cell adhesion of epithelial cells [42,43].

To determine whether lack of APC affected the distribution of other proteins normally restricted to specific membrane compartments, we investigated the localization of the apical marker ezrin in organoids. Ezrin plays a role in the interaction of the brush border actin cytoskeleton and the plasma membrane [21,22]. Loss of APC had no effect on the apical accumulation of ezrin suggesting that its association with the actin cytoskeleton was intact (figure 4*e,f*). To further characterize the polarity of cells, we stained organoids for Na/K ATPase (figure 5*a,b*). The restriction of Na^+^/K^+^ATPase to the lateral membrane was found in both wild-type and APC*^fl/fl^* organoids. Interestingly, the levels of Na^+^/K^+^ATPase were higher in differentiated villus-like domains when compared with crypt-like domains in wild-type organoids. Other than its confirmed expression in gut epithelium, not much is known about the differential expression of the Na^+^/K^+^ATPase along the crypt–villus axis [44,45]. It is possible that cells in the villus require more of the Na^+^/K^+^ATPase to maintain the electrochemical gradient because they are challenged by direct exposure to gut contents and the absorption of nutrients and ions. Cells in the crypt may be sheltered by their position in the invaginated crypt, which permits accumulation of the viscous mucus layer produced by secretory cells and shields them from the effect of gut contents.

The level of Na^+^/K^+^ATPase in the APC^*fl/fl*^ appeared low and comparable with that in the crypt-like domains of wild-type organoids, consistent with the idea that cysts represent crypt-like regions of the epithelium. This is supported by our observation that almost all the cells in the APC*^fl/fl^* organoids were proliferative. Finally, we investigated the distribution of a protein in organoids that facilities their interaction with the surrounding ECM. We determined the localization of the β4 integrin, expressed in cells of the intestine to link them to laminin in the ECM [36–38]. In both wild-type and APC*^fl/fl^* organoids, β4 integrin localized specifically to basal membranes where cells interact with the ECM.

In summary, the distributions of a variety of cell polarity markers were similar in both wild-type and APC*^fl/fl^* organoids consistent with the idea that cell polarity is maintained in the absence of APC despite a remarkable loss of tissue polarity. It is possible that cell polarity is lost only after further mutations have occurred subsequent to APC loss.

Despite continued apical–basal polarity in APC*^fl/fl^* organoids, they exhibited some notable changes in epithelial organization. There was a larger variation in the size and shape of cells. Cells in cysts were flatter and less elongated along their apical–basal axis. In some cases, cells in cysts piled up to form multilayered structures in the cyst wall. Taken together, these observations indicate that although cell–cell adhesion *per se* remained intact in the absence of APC, cytoskeletal organization was altered with shorter microtubule arrays and a broader, less developed brush borders. Additionally, the wall of APC*^fl/fl^* organoids cysts often formed furrow-like invaginations that were the result of collapsed cyst walls. This could be the consequence of changes in the mechanical properties of cells and the resulting tissue. A change in mechanical properties could also contribute to changes in tissue shape to cause the spheroid structures instead of the highly curved crypt bases adjacent to an elongated crypt neck. Alternatively or in addition, the lower expression of the Na^+^/K^+^ATPase in the APC*^fl/fl^* organoids may not provide sufficient ion exchange to maintain the hydrostatic pressure required to keep organoids ‘inflated’.

It is not clear whether the inability to organize a normal tissue axis after loss of APC is due to the lack of differentiation and increased proliferation and/or whether changes in cytoskeletal regulation and other processes that loss of normal APC is predicted to cause are to blame [46]. The fact that we did not observe an increase in the nuclear localization of β-catenin in APC*^fl/fl^* organoids suggests that although there may be some increase in the transcriptional activation mediated by β-catenin, the hyperactivation of the transcriptional activity of β-catenin that is normally used to explain proliferative changes in APC mutant cells is likely not the only driving force for these changes. Instead, it is highly likely that changes in both β-catenin-related and non-related APC functions are responsible. The overall effect is an inability of cells to create the tissue organization required to perform normal function to maintain homeostasis.

Using intestinal organoids to determine the link between specific molecular events and resulting changes in tissue organization during cancer progression will reveal how different elements of cell and tissue polarity are linked and how tumourigenic mutations exploit these links to drive transformation. This will not only inform about disease processes it also creates a potentially powerful tool for developing and testing new therapeutics.
